# 21-(4-Methyl­phenyl­sulfon­yl)-4,7,13,16-tetra­oxa-1,10,21-triaza­bicyclo­[8.8.5]tricosane-19,23-dione: an *N*-tosyl­ated macrobicyclic dilactam

**DOI:** 10.1107/S1600536811018873

**Published:** 2011-05-28

**Authors:** Trevor K. Ellis, Stephen M. Clayton, Douglas R. Powell, Richard W. Taylor

**Affiliations:** a University of Oklahoma, Department of Chemistry and Biochemistry, 101 Stephenson Pkwy, Norman, OK 73019-5251, USA

## Abstract

The macrobicyclic title compound, C_23_H_35_N_3_O_8_S, contains two tertiary amide bridgehead N atoms and a toluene­sulfonamide N atom in the center of the five-atom bridging strand. The mol­ecule has a central cavity that is defined by the 18-membered ring identified by the N_2_O_4_ donor atom set and two 15-membered rings with N_3_O_2_ donor atom sets. The toluene­sulfonamide N atom adopts an *exo* orientation with respect to the central cavity, and the tosyl group is oriented on one side of the aza-bridging strand that connects the bridgehead N atoms.

## Related literature

For general background to bicyclic dilactams as cation receptors, see: Hourdakis & Popov (1977 ▶); Tümmler *et al.* (1977 ▶); Buschmann, (1986 ▶); Pietraszkiewicz *et al.* (1992 ▶); Wanichacheva *et al.* (2006*a* ▶,*b* ▶). For related structures, see: Fields *et al.* (1986 ▶); Tarnowska *et al.* (2004 ▶). For the synthesis, see: Lehn & Montavon (1976 ▶, 1978 ▶); Lehn *et al.* (1977 ▶); Frère & Gramain (1982 ▶); Pietraszkiewicz *et al.* (1992 ▶); Wanichacheva *et al.* (2006*a* ▶,*b* ▶).
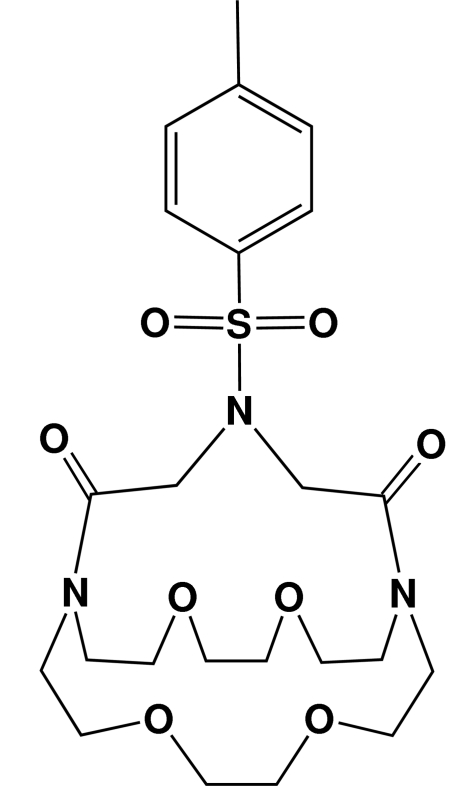


## Experimental

### 

#### Crystal data

C_23_H_35_N_3_O_8_S
*M* *_r_* = 513.60
Monoclinic, 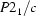

*a* = 12.807 (2) Å
*b* = 20.096 (3) Å
*c* = 10.3305 (17) Å
β = 112.949 (3)°
*V* = 2448.3 (7) Å^3^
*Z* = 4
Mo *K*α radiation
μ = 0.19 mm^−1^
*T* = 100 K
0.45 × 0.34 × 0.02 mm

#### Data collection

Bruker APEX CCD diffractometer
Absorption correction: multi-scan (*SADABS*; Sheldrick, 2001 ▶) *T*_min_ = 0.921, *T*_max_ = 0.996
22174 measured reflections
5330 independent reflections
3651 reflections with *I* > 2σ(*I*)
*R*_int_ = 0.078

#### Refinement

*R*[*F* ^2^ > 2σ(*F* ^2^)] = 0.056
*wR*(*F* ^2^) = 0.152
*S* = 1.00
5330 reflections
316 parameters
H-atom parameters constrained
Δρ_max_ = 0.87 e Å^−3^
Δρ_min_ = −0.74 e Å^−3^

### 

Data collection: *SMART* (Bruker, 2007 ▶); cell refinement: *SAINT* (Bruker, 2007 ▶); data reduction: *SAINT*; program(s) used to solve structure: *SHELXTL* (Sheldrick, 2008 ▶); program(s) used to refine structure: *SHELXTL*; molecular graphics: *SHELXTL*; software used to prepare material for publication: *SHELXTL*.

## Supplementary Material

Crystal structure: contains datablocks I, global. DOI: 10.1107/S1600536811018873/pk2323sup1.cif

Structure factors: contains datablocks I. DOI: 10.1107/S1600536811018873/pk2323Isup2.hkl

Supplementary material file. DOI: 10.1107/S1600536811018873/pk2323Isup3.cml

Additional supplementary materials:  crystallographic information; 3D view; checkCIF report

## Figures and Tables

**Fig. 1.**
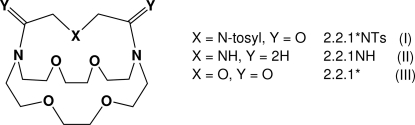
Structural formulae of the title compound (I) and related 2.2.1-type cryptands.

**Fig. 2.**
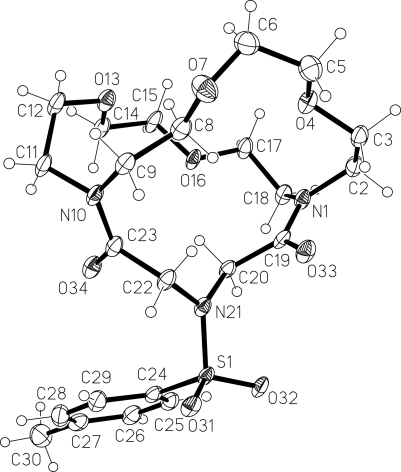
The molecular structure of the title compound. Displacement ellipsoids are drawn at the 50% probability level.

## supplementary crystallographic information

### Comment

The title compound (I) was isolated as an intermediate in the synthesis of the corresponding azacryptand, 2.2.1_NH_ (II, see Figure 1). An analogous bicyclic diamide, 2.2.1* (III), containing only oxygen donor atoms in the bridging strands, has been reported (Tarnowska, *et al.*, 2004). Cryptands where one or more O atoms have been replaced by N atoms are of interest because of their selectivity for transition- and heavy-metal cations (Lehn & Montavon, 1978). In addition, the nitrogen atom may serve as a point of attachment for sensor chromophores (Wanichacheva, *et al.*, 2006*a*) or cryptand-based polymer resins (Frère & Gramain, 1982). The bicyclic 2.2.1*_NR_ dilactam with *R* = (CH_2_)_9_CH_3_ (Pietraszkiewicz *et al.*, 1992) or *R* = –C(=O)(CH_2_)_15_SCH_3_ (Wanichacheva, *et al.*, 2006*b*) has been used in the construction of ion selective electrodes.

Figure 2 shows that (I) consists of an 18-membered ring (donor atoms N1, O4, O7, N10, O13, O16) and two 15-membered rings with N_3_O_2_ donor atom sets (N1, O4, O7, N21, N10 and N1, O13, O16, N10, N21). With respect to the molecular cavity formed by these rings, donor atoms N1, O3, O13, O16 and N10 have an endodentate orientation, while N21, O7 and the carbonyl O atoms, O33 and O34, are exodentate. The oxygen donor atoms of the 18-membered ring (O4, O7, O13, O16) form a plane (average deviation = 0.314 Å) that is nearly perpendicular to the plane defined by nitrogen atoms N1, N10, N21 (dihedral angle = 83.7 (3)°). The toluenesulfonamide group is oriented over the face of one 15-membered ring (N1, O13, O16, N10, N21) and the plane of the benzene ring (C24—C29), average deviation = 0.0042 Å) is almost coplanar with the oxygen donor plane defined by O4, O7, O13, O16 (dihedral angle = 16.5 (3)°). The N1···N10 nonbonding distance is 5.299 (4)Å which is less than the value of 5.643 (4)Å found for 2.2.1* (Tarnowska *et al.*, 2004). The aza bridging strand consists of three planar subunits: N1, C19, O33, C20, average deviation = 0.0021 Å; C20, N21, S1, C22, average deviation = 0.0255 Å; and C22, C23, O34, N10, average deviation = 0.0068 Å. The limited conformational freedom of this bridge may explain the shorter N1···N10 nonbonding distance compared to the more flexible 2.2.1* and the extensive splitting in the ^1^H-NMR spectrum due to the non-equivalence of the methylene protons.

### Experimental

The bicyclic diamide was obtained by the high-dilution condensation of 1,10-diaza-4,7,13,16-tetraoxacyclooctadecane with 2,2'-(*N*-tosyl)diacetyl chloride according to reported methods (Lehn & Montavon, 1976). The *N*-protected diacid chloride was prepared following literature procedures (Lehn, *et al.*, 1977). The crude dilactam was purified by flash column chromatography on silica gel using a mixture of CHCl_3_ and acetone (4:1) as the eluent. Spectroscopic Analysis: ^1^H-NMR (CDCl_3_, 300 MHz) δ 2.38 (s, 3H), δ 2.57–2.64 (ddd, 2H), δ 2.90–3.00 (ddd, 2H), δ 3.49–3.65 (m, 10H), δ 3.67–3.80 (m, 8H), δ 4.36 (d, 4H), δ 4.53 (ddd, 2H), δ 7.25, 7.90 (q, 4H); ESI-MS: m/*z* = 536.2 (*M* + Na^+^) and 1049.5 (2*M* + Na^+^). Crystals suitable for X-ray crystallography were obtained by slow evaporation of a solution of the compound dissolved in toluene-methanol (1:1).

### Refinement

H atoms were positioned geometrically and refined using a riding model with C—H = 0.95Å for aromatic carbons, 0.98Å for methyl carbons, and 0.99Å for methylene carbons.  *U*_iso_(H) = 1.2 *U*_eq_(C) or 1.5 *U*_eq_(C_Me_).

### Crystal data

**Table:**

C_23_H_35_N_3_O_8_S	*F*(000) = 1096
*M**_r_* = 513.60	*D*_x_ = 1.393 Mg m^−^^3^
Monoclinic, *P*2_1_/*c*	Mo *K*α radiation, λ = 0.71073 Å
Hall symbol: -P 2ybc	Cell parameters from 7056 reflections
*a* = 12.807 (2) Å	θ = 2.4–26.8°
*b* = 20.096 (3) Å	µ = 0.19 mm^−^^1^
*c* = 10.3305 (17) Å	*T* = 100 K
β = 112.949 (3)°	Plate, colorless
*V* = 2448.3 (7) Å^3^	0.45 × 0.34 × 0.02 mm
*Z* = 4	

### Data collection

**Table:**

Bruker APEX CCD diffractometer	5330 independent reflections
Radiation source: fine-focus sealed tube	3651 reflections with *I* > 2σ(*I*)
graphite	*R*_int_ = 0.078
ω scans	θ_max_ = 27.0°, θ_min_ = 2.0°
Absorption correction: multi-scan (*SADABS*; Sheldrick, 2001)	*h* = −16→16
*T*_min_ = 0.921, *T*_max_ = 0.996	*k* = −25→25
22174 measured reflections	*l* = −13→13

### Refinement

**Table:**

Refinement on *F*^2^	Primary atom site location: structure-invariant direct methods
Least-squares matrix: full	Secondary atom site location: difference Fourier map
*R*[*F*^2^ > 2σ(*F*^2^)] = 0.056	Hydrogen site location: inferred from neighbouring sites
*wR*(*F*^2^) = 0.152	H-atom parameters constrained
*S* = 1.00	*w* = 1/[σ^2^(*F*_o_^2^) + (0.090*P*)^2^ + ] where *P* = (*F*_o_^2^ + 2*F*_c_^2^)/3
5330 reflections	(Δ/σ)_max_ = 0.001
316 parameters	Δρ_max_ = 0.87 e Å^−^^3^
0 restraints	Δρ_min_ = −0.74 e Å^−^^3^

### Fractional atomic coordinates and isotropic or equivalent isotropic displacement parameters (Å^2^)

**Table:**

	*x*	*y*	*z*	*U*_iso_*/*U*_eq_	
S1	1.09333 (5)	0.25045 (3)	0.83451 (6)	0.01752 (16)	
O31	1.09605 (15)	0.22025 (8)	0.71044 (17)	0.0212 (4)	
O32	1.08925 (14)	0.20910 (8)	0.94505 (17)	0.0213 (4)	
O33	0.79170 (15)	0.24397 (8)	0.79188 (18)	0.0237 (4)	
O34	1.03764 (15)	0.42449 (8)	0.72294 (18)	0.0233 (4)	
N1	0.77810 (17)	0.31622 (9)	0.9521 (2)	0.0190 (4)	
C2	0.6686 (2)	0.28984 (12)	0.9424 (3)	0.0235 (6)	
H2A	0.6390	0.3198	0.9962	0.028*	
H2B	0.6823	0.2460	0.9897	0.028*	
C3	0.5777 (2)	0.28131 (12)	0.7980 (3)	0.0232 (6)	
H3A	0.5988	0.2448	0.7484	0.028*	
H3B	0.5054	0.2691	0.8057	0.028*	
O4	0.56315 (14)	0.34114 (8)	0.72001 (18)	0.0247 (4)	
C5	0.4764 (2)	0.33533 (14)	0.5853 (3)	0.0296 (6)	
H5A	0.4018	0.3350	0.5931	0.036*	
H5B	0.4850	0.2928	0.5419	0.036*	
C6	0.4817 (2)	0.39256 (15)	0.4942 (3)	0.0322 (7)	
H6A	0.4072	0.3971	0.4149	0.039*	
H6B	0.4961	0.4341	0.5499	0.039*	
O7	0.56706 (15)	0.38508 (10)	0.43936 (19)	0.0326 (5)	
C8	0.6766 (2)	0.40118 (12)	0.5372 (3)	0.0240 (6)	
H8A	0.7052	0.3655	0.6084	0.029*	
H8B	0.6745	0.4433	0.5859	0.029*	
C9	0.7540 (2)	0.40869 (12)	0.4576 (3)	0.0215 (5)	
H9A	0.7651	0.3647	0.4217	0.026*	
H9B	0.7177	0.4384	0.3759	0.026*	
N10	0.86434 (17)	0.43611 (9)	0.5476 (2)	0.0200 (4)	
C11	0.8809 (2)	0.50799 (11)	0.5374 (3)	0.0230 (6)	
H11A	0.8792	0.5180	0.4428	0.028*	
H11B	0.9570	0.5203	0.6070	0.028*	
C12	0.7929 (2)	0.55061 (12)	0.5623 (3)	0.0244 (6)	
H12A	0.8161	0.5978	0.5666	0.029*	
H12B	0.7198	0.5457	0.4808	0.029*	
O13	0.77576 (15)	0.53522 (8)	0.68612 (17)	0.0226 (4)	
C14	0.8673 (2)	0.55091 (12)	0.8130 (3)	0.0239 (6)	
H14A	0.8822	0.5994	0.8183	0.029*	
H14B	0.9367	0.5275	0.8177	0.029*	
C15	0.8367 (2)	0.52969 (11)	0.9315 (3)	0.0236 (6)	
H15A	0.8870	0.5517	1.0194	0.028*	
H15B	0.7574	0.5426	0.9125	0.028*	
O16	0.84885 (15)	0.45891 (8)	0.94636 (17)	0.0215 (4)	
C17	0.8009 (2)	0.43394 (11)	1.0383 (3)	0.0229 (5)	
H17A	0.7174	0.4381	0.9943	0.027*	
H17B	0.8286	0.4601	1.1265	0.027*	
C18	0.8337 (2)	0.36153 (11)	1.0704 (3)	0.0207 (5)	
H18A	0.9168	0.3574	1.1002	0.025*	
H18B	0.8146	0.3474	1.1504	0.025*	
C19	0.8310 (2)	0.29040 (11)	0.8733 (2)	0.0192 (5)	
C20	0.9430 (2)	0.32263 (11)	0.8889 (2)	0.0180 (5)	
H20A	0.9327	0.3714	0.8783	0.022*	
H20B	1.0005	0.3135	0.9842	0.022*	
N21	0.98335 (17)	0.29775 (9)	0.7851 (2)	0.0180 (4)	
C22	0.9321 (2)	0.32465 (11)	0.6440 (2)	0.0195 (5)	
H22A	0.8500	0.3144	0.6043	0.023*	
H22B	0.9664	0.3031	0.5838	0.023*	
C23	0.9485 (2)	0.40027 (12)	0.6426 (2)	0.0197 (5)	
C24	1.2164 (2)	0.29981 (11)	0.9071 (3)	0.0189 (5)	
C25	1.2716 (2)	0.30417 (12)	1.0509 (3)	0.0219 (5)	
H25	1.2430	0.2814	1.1107	0.026*	
C26	1.3687 (2)	0.34197 (12)	1.1071 (3)	0.0251 (6)	
H26	1.4071	0.3450	1.2063	0.030*	
C27	1.4114 (2)	0.37568 (12)	1.0212 (3)	0.0254 (6)	
C28	1.3541 (2)	0.37067 (12)	0.8774 (3)	0.0278 (6)	
H28	1.3826	0.3936	0.8176	0.033*	
C29	1.2567 (2)	0.33334 (12)	0.8185 (3)	0.0238 (6)	
H29	1.2180	0.3306	0.7193	0.029*	
C30	1.5165 (2)	0.41723 (14)	1.0863 (3)	0.0348 (7)	
H30A	1.5386	0.4349	1.0123	0.052*	
H30B	1.5780	0.3897	1.1507	0.052*	
H30C	1.5014	0.4542	1.1385	0.052*	

### Atomic displacement parameters (Å^2^)

**Table:**

	*U*^11^	*U*^22^	*U*^33^	*U*^12^	*U*^13^	*U*^23^
S1	0.0290 (3)	0.0091 (3)	0.0181 (3)	0.0008 (2)	0.0132 (3)	−0.0004 (2)
O31	0.0373 (10)	0.0126 (8)	0.0184 (9)	0.0015 (7)	0.0160 (8)	−0.0042 (7)
O32	0.0362 (10)	0.0119 (8)	0.0200 (9)	0.0016 (7)	0.0155 (8)	0.0034 (7)
O33	0.0359 (10)	0.0133 (8)	0.0255 (10)	−0.0022 (7)	0.0158 (8)	−0.0042 (7)
O34	0.0326 (10)	0.0158 (9)	0.0217 (9)	−0.0019 (7)	0.0108 (8)	−0.0001 (7)
N1	0.0296 (11)	0.0125 (9)	0.0193 (11)	−0.0011 (8)	0.0143 (9)	−0.0009 (8)
C2	0.0327 (14)	0.0172 (12)	0.0270 (14)	0.0007 (10)	0.0186 (12)	0.0030 (10)
C3	0.0315 (14)	0.0138 (12)	0.0304 (14)	−0.0014 (10)	0.0188 (12)	−0.0003 (10)
O4	0.0329 (10)	0.0184 (9)	0.0236 (9)	−0.0005 (7)	0.0119 (8)	0.0039 (7)
C5	0.0305 (14)	0.0334 (15)	0.0270 (15)	−0.0026 (12)	0.0134 (12)	−0.0004 (12)
C6	0.0277 (14)	0.0426 (17)	0.0285 (15)	0.0059 (12)	0.0134 (12)	0.0094 (13)
O7	0.0289 (10)	0.0479 (13)	0.0237 (10)	−0.0007 (9)	0.0131 (8)	0.0017 (9)
C8	0.0320 (14)	0.0182 (12)	0.0228 (14)	0.0010 (10)	0.0119 (11)	0.0004 (10)
C9	0.0287 (13)	0.0159 (12)	0.0204 (13)	0.0017 (10)	0.0099 (11)	0.0023 (10)
N10	0.0296 (11)	0.0105 (9)	0.0223 (11)	0.0007 (8)	0.0127 (9)	0.0024 (8)
C11	0.0365 (15)	0.0117 (11)	0.0246 (14)	0.0013 (10)	0.0161 (12)	0.0053 (10)
C12	0.0400 (15)	0.0124 (11)	0.0245 (14)	0.0022 (10)	0.0168 (12)	0.0045 (10)
O13	0.0340 (10)	0.0158 (8)	0.0212 (9)	0.0001 (7)	0.0142 (8)	0.0000 (7)
C14	0.0351 (15)	0.0114 (11)	0.0267 (14)	−0.0007 (10)	0.0137 (12)	0.0016 (10)
C15	0.0405 (15)	0.0077 (11)	0.0252 (14)	−0.0001 (10)	0.0156 (12)	−0.0003 (9)
O16	0.0379 (10)	0.0083 (8)	0.0264 (10)	0.0010 (7)	0.0213 (8)	0.0004 (7)
C17	0.0378 (15)	0.0130 (11)	0.0265 (14)	−0.0007 (10)	0.0221 (12)	−0.0006 (10)
C18	0.0321 (14)	0.0152 (11)	0.0199 (13)	0.0001 (10)	0.0157 (11)	−0.0003 (9)
C19	0.0310 (14)	0.0102 (11)	0.0188 (13)	0.0041 (9)	0.0121 (11)	0.0042 (9)
C20	0.0285 (13)	0.0114 (11)	0.0177 (12)	0.0027 (9)	0.0129 (10)	0.0004 (9)
N21	0.0296 (11)	0.0134 (9)	0.0152 (10)	0.0022 (8)	0.0132 (9)	0.0014 (8)
C22	0.0311 (13)	0.0125 (11)	0.0167 (12)	0.0011 (9)	0.0112 (11)	−0.0005 (9)
C23	0.0340 (14)	0.0143 (11)	0.0165 (12)	0.0031 (10)	0.0162 (11)	0.0017 (9)
C24	0.0268 (13)	0.0113 (11)	0.0214 (13)	0.0034 (9)	0.0123 (11)	0.0001 (9)
C25	0.0301 (14)	0.0159 (12)	0.0225 (13)	0.0032 (10)	0.0134 (11)	0.0007 (10)
C26	0.0335 (14)	0.0186 (12)	0.0242 (14)	0.0028 (11)	0.0124 (12)	0.0008 (10)
C27	0.0294 (14)	0.0155 (12)	0.0326 (15)	0.0022 (10)	0.0136 (12)	−0.0038 (11)
C28	0.0378 (15)	0.0202 (13)	0.0333 (16)	−0.0015 (11)	0.0225 (13)	0.0001 (11)
C29	0.0339 (14)	0.0193 (12)	0.0224 (13)	0.0002 (10)	0.0155 (12)	−0.0005 (10)
C30	0.0361 (16)	0.0299 (15)	0.0413 (18)	−0.0059 (12)	0.0181 (14)	−0.0068 (13)

### Geometric parameters (Å, °)

**Table:**

S1—O32	1.4295 (16)	C12—H12B	0.9900
S1—O31	1.4308 (16)	O13—C14	1.412 (3)
S1—N21	1.608 (2)	C14—C15	1.486 (3)
S1—C24	1.763 (2)	C14—H14A	0.9900
O33—C19	1.226 (3)	C14—H14B	0.9900
O34—C23	1.220 (3)	C15—O16	1.433 (3)
N1—C19	1.349 (3)	C15—H15A	0.9900
N1—C2	1.465 (3)	C15—H15B	0.9900
N1—C18	1.467 (3)	O16—C17	1.408 (3)
C2—C3	1.502 (4)	C17—C18	1.515 (3)
C2—H2A	0.9900	C17—H17A	0.9900
C2—H2B	0.9900	C17—H17B	0.9900
C3—O4	1.418 (3)	C18—H18A	0.9900
C3—H3A	0.9900	C18—H18B	0.9900
C3—H3B	0.9900	C19—C20	1.523 (3)
O4—C5	1.406 (3)	C20—N21	1.449 (3)
C5—C6	1.505 (4)	C20—H20A	0.9900
C5—H5A	0.9900	C20—H20B	0.9900
C5—H5B	0.9900	N21—C22	1.450 (3)
C6—O7	1.421 (3)	C22—C23	1.535 (3)
C6—H6A	0.9900	C22—H22A	0.9900
C6—H6B	0.9900	C22—H22B	0.9900
O7—C8	1.410 (3)	C24—C25	1.377 (3)
C8—C9	1.523 (3)	C24—C29	1.388 (3)
C8—H8A	0.9900	C25—C26	1.378 (4)
C8—H8B	0.9900	C25—H25	0.9500
C9—N10	1.463 (3)	C26—C27	1.386 (4)
C9—H9A	0.9900	C26—H26	0.9500
C9—H9B	0.9900	C27—C28	1.380 (4)
N10—C23	1.349 (3)	C27—C30	1.502 (4)
N10—C11	1.470 (3)	C28—C29	1.377 (4)
C11—C12	1.516 (3)	C28—H28	0.9500
C11—H11A	0.9900	C29—H29	0.9500
C11—H11B	0.9900	C30—H30A	0.9800
C12—O13	1.414 (3)	C30—H30B	0.9800
C12—H12A	0.9900	C30—H30C	0.9800
			
O32—S1—O31	119.36 (10)	C15—C14—H14B	110.1
O32—S1—N21	107.27 (10)	H14A—C14—H14B	108.4
O31—S1—N21	106.89 (10)	O16—C15—C14	108.70 (19)
O32—S1—C24	107.00 (11)	O16—C15—H15A	109.9
O31—S1—C24	106.83 (11)	C14—C15—H15A	109.9
N21—S1—C24	109.24 (11)	O16—C15—H15B	109.9
C19—N1—C2	120.8 (2)	C14—C15—H15B	109.9
C19—N1—C18	123.1 (2)	H15A—C15—H15B	108.3
C2—N1—C18	114.95 (19)	C17—O16—C15	111.61 (17)
N1—C2—C3	117.4 (2)	O16—C17—C18	109.52 (19)
N1—C2—H2A	108.0	O16—C17—H17A	109.8
C3—C2—H2A	108.0	C18—C17—H17A	109.8
N1—C2—H2B	108.0	O16—C17—H17B	109.8
C3—C2—H2B	108.0	C18—C17—H17B	109.8
H2A—C2—H2B	107.2	H17A—C17—H17B	108.2
O4—C3—C2	110.3 (2)	N1—C18—C17	114.2 (2)
O4—C3—H3A	109.6	N1—C18—H18A	108.7
C2—C3—H3A	109.6	C17—C18—H18A	108.7
O4—C3—H3B	109.6	N1—C18—H18B	108.7
C2—C3—H3B	109.6	C17—C18—H18B	108.7
H3A—C3—H3B	108.1	H18A—C18—H18B	107.6
C5—O4—C3	111.67 (19)	O33—C19—N1	122.5 (2)
O4—C5—C6	110.2 (2)	O33—C19—C20	120.9 (2)
O4—C5—H5A	109.6	N1—C19—C20	116.5 (2)
C6—C5—H5A	109.6	N21—C20—C19	111.60 (19)
O4—C5—H5B	109.6	N21—C20—H20A	109.3
C6—C5—H5B	109.6	C19—C20—H20A	109.3
H5A—C5—H5B	108.1	N21—C20—H20B	109.3
O7—C6—C5	113.4 (2)	C19—C20—H20B	109.3
O7—C6—H6A	108.9	H20A—C20—H20B	108.0
C5—C6—H6A	108.9	C20—N21—C22	117.56 (18)
O7—C6—H6B	108.9	C20—N21—S1	119.35 (16)
C5—C6—H6B	108.9	C22—N21—S1	122.47 (16)
H6A—C6—H6B	107.7	N21—C22—C23	111.63 (19)
C8—O7—C6	113.3 (2)	N21—C22—H22A	109.3
O7—C8—C9	108.1 (2)	C23—C22—H22A	109.3
O7—C8—H8A	110.1	N21—C22—H22B	109.3
C9—C8—H8A	110.1	C23—C22—H22B	109.3
O7—C8—H8B	110.1	H22A—C22—H22B	108.0
C9—C8—H8B	110.1	O34—C23—N10	123.5 (2)
H8A—C8—H8B	108.4	O34—C23—C22	118.9 (2)
N10—C9—C8	111.4 (2)	N10—C23—C22	117.5 (2)
N10—C9—H9A	109.3	C25—C24—C29	120.9 (2)
C8—C9—H9A	109.3	C25—C24—S1	119.50 (19)
N10—C9—H9B	109.3	C29—C24—S1	119.57 (19)
C8—C9—H9B	109.3	C24—C25—C26	119.3 (2)
H9A—C9—H9B	108.0	C24—C25—H25	120.4
C23—N10—C9	124.1 (2)	C26—C25—H25	120.4
C23—N10—C11	118.7 (2)	C25—C26—C27	121.1 (2)
C9—N10—C11	117.10 (19)	C25—C26—H26	119.5
N10—C11—C12	113.9 (2)	C27—C26—H26	119.5
N10—C11—H11A	108.8	C28—C27—C26	118.5 (2)
C12—C11—H11A	108.8	C28—C27—C30	122.0 (2)
N10—C11—H11B	108.8	C26—C27—C30	119.6 (2)
C12—C11—H11B	108.8	C29—C28—C27	121.7 (2)
H11A—C11—H11B	107.7	C29—C28—H28	119.2
O13—C12—C11	114.7 (2)	C27—C28—H28	119.2
O13—C12—H12A	108.6	C28—C29—C24	118.6 (2)
C11—C12—H12A	108.6	C28—C29—H29	120.7
O13—C12—H12B	108.6	C24—C29—H29	120.7
C11—C12—H12B	108.6	C27—C30—H30A	109.5
H12A—C12—H12B	107.6	C27—C30—H30B	109.5
C14—O13—C12	115.17 (19)	H30A—C30—H30B	109.5
O13—C14—C15	108.1 (2)	C27—C30—H30C	109.5
O13—C14—H14A	110.1	H30A—C30—H30C	109.5
C15—C14—H14A	110.1	H30B—C30—H30C	109.5
O13—C14—H14B	110.1		
			
C19—N1—C2—C3	−49.0 (3)	O32—S1—N21—C20	36.3 (2)
C18—N1—C2—C3	142.7 (2)	O31—S1—N21—C20	165.43 (17)
N1—C2—C3—O4	−51.6 (3)	C24—S1—N21—C20	−79.33 (19)
C2—C3—O4—C5	−178.5 (2)	O32—S1—N21—C22	−152.89 (18)
C3—O4—C5—C6	−166.4 (2)	O31—S1—N21—C22	−23.8 (2)
O4—C5—C6—O7	78.8 (3)	C24—S1—N21—C22	91.5 (2)
C5—C6—O7—C8	−79.4 (3)	C20—N21—C22—C23	59.8 (3)
C6—O7—C8—C9	−165.6 (2)	S1—N21—C22—C23	−111.2 (2)
O7—C8—C9—N10	170.55 (19)	C9—N10—C23—O34	−175.5 (2)
C8—C9—N10—C23	79.0 (3)	C11—N10—C23—O34	1.3 (4)
C8—C9—N10—C11	−97.8 (2)	C9—N10—C23—C22	7.2 (3)
C23—N10—C11—C12	−121.6 (2)	C11—N10—C23—C22	−176.0 (2)
C9—N10—C11—C12	55.4 (3)	N21—C22—C23—O34	36.9 (3)
N10—C11—C12—O13	50.6 (3)	N21—C22—C23—N10	−145.7 (2)
C11—C12—O13—C14	68.8 (3)	O32—S1—C24—C25	−14.6 (2)
C12—O13—C14—C15	−178.22 (18)	O31—S1—C24—C25	−143.47 (19)
O13—C14—C15—O16	76.8 (2)	N21—S1—C24—C25	101.2 (2)
C14—C15—O16—C17	−168.7 (2)	O32—S1—C24—C29	164.98 (18)
C15—O16—C17—C18	−170.2 (2)	O31—S1—C24—C29	36.1 (2)
C19—N1—C18—C17	103.2 (3)	N21—S1—C24—C29	−79.2 (2)
C2—N1—C18—C17	−88.9 (2)	C29—C24—C25—C26	−0.5 (4)
O16—C17—C18—N1	−70.9 (3)	S1—C24—C25—C26	178.99 (18)
C2—N1—C19—O33	−0.6 (4)	C24—C25—C26—C27	0.2 (4)
C18—N1—C19—O33	166.6 (2)	C25—C26—C27—C28	0.1 (4)
C2—N1—C19—C20	178.53 (19)	C25—C26—C27—C30	179.1 (2)
C18—N1—C19—C20	−14.2 (3)	C26—C27—C28—C29	0.0 (4)
O33—C19—C20—N21	7.8 (3)	C30—C27—C28—C29	−178.9 (2)
N1—C19—C20—N21	−171.41 (19)	C27—C28—C29—C24	−0.4 (4)
C19—C20—N21—C22	79.0 (2)	C25—C24—C29—C28	0.7 (4)
C19—C20—N21—S1	−109.7 (2)	S1—C24—C29—C28	−178.89 (19)
